# 2-[(Pyrimidin-2-yl­amino)­meth­yl]phenol

**DOI:** 10.1107/S1600536811046848

**Published:** 2011-11-12

**Authors:** Jing Xu, Shan Gao, Seik Weng Ng

**Affiliations:** a Key Laboratory of Functional Inorganic Material Chemistry, Ministry of Education, Heilongjiang University, Harbin 150080, People’s Republic of China; b Department of Chemistry, University of Malaya, 50603 Kuala Lumpur, Malaysia; c Chemistry Department, Faculty of Science, King Abdulaziz University, PO Box 80203 Jeddah, Saudi Arabia

## Abstract

In the title compound, C_11_H_11_N_3_O, the aromatic rings at either ends of the –CH_2_–NH– link are twisted by 72.58 (8)°; the hy­droxy substituent is a hydrogen-bond donor to an N atom of the pyrimidine ring. The other N atom of the pyrimidine ring is a hydrogen-bond acceptor to the amino group of an inversion-related mol­ecule.

## Related literature

For the *N*-salicyl­idene-2-amino­pyrimidine precursor, see: El-Haty *et al.* (1990 ▶); Issa *et al.* (2011 ▶); Shalabi & Abdel-Ghani (1990 ▶). For a related structure, see: Xu *et al.* (2011 ▶).
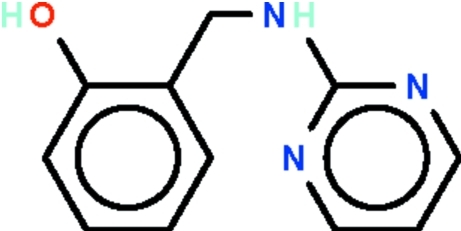


## Experimental

### 

#### Crystal data

C_11_H_11_N_3_O
*M* *_r_* = 201.23
Monoclinic, 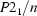

*a* = 5.8625 (4) Å
*b* = 9.3610 (7) Å
*c* = 18.4058 (13) Å
β = 95.208 (2)°
*V* = 1005.92 (12) Å^3^
*Z* = 4
Mo *K*α radiation
μ = 0.09 mm^−1^
*T* = 293 K
0.22 × 0.17 × 0.15 mm

#### Data collection

Rigaku R-AXIS RAPID IP diffractometer
Absorption correction: multi-scan (*ABSCOR*; Higashi, 1995 ▶) *T*_min_ = 0.981, *T*_max_ = 0.987
9626 measured reflections
2296 independent reflections
1476 reflections with *I* > 2σ(*I*)
*R*_int_ = 0.030

#### Refinement

*R*[*F* ^2^ > 2σ(*F* ^2^)] = 0.040
*wR*(*F* ^2^) = 0.149
*S* = 1.12
2296 reflections
144 parameters
2 restraints
H atoms treated by a mixture of independent and constrained refinement
Δρ_max_ = 0.14 e Å^−3^
Δρ_min_ = −0.15 e Å^−3^

### 

Data collection: *RAPID-AUTO* (Rigaku, 1998 ▶); cell refinement: *RAPID-AUTO*; data reduction: *CrystalClear* (Rigaku/MSC, 2002 ▶); program(s) used to solve structure: *SHELXS97* (Sheldrick, 2008 ▶); program(s) used to refine structure: *SHELXL97* (Sheldrick, 2008 ▶); molecular graphics: *X-SEED* (Barbour, 2001 ▶); software used to prepare material for publication: *publCIF* (Westrip, 2010 ▶).

## Supplementary Material

Crystal structure: contains datablock(s) global, I. DOI: 10.1107/S1600536811046848/xu5383sup1.cif

Structure factors: contains datablock(s) I. DOI: 10.1107/S1600536811046848/xu5383Isup2.hkl

Supplementary material file. DOI: 10.1107/S1600536811046848/xu5383Isup3.cml

Additional supplementary materials:  crystallographic information; 3D view; checkCIF report

## Figures and Tables

**Table 1:** Hydrogen-bond geometry (Å, °)

*D*—H⋯*A*	*D*—H	H⋯*A*	*D*⋯*A*	*D*—H⋯*A*
O1—H1o⋯N2	0.86 (1)	1.92 (1)	2.761 (2)	164 (2)
N1—H1n⋯N3^i^	0.88 (1)	2.15 (1)	3.023 (2)	176 (2)

Symmetry code: (i) 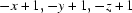.

**Fig. 1.**
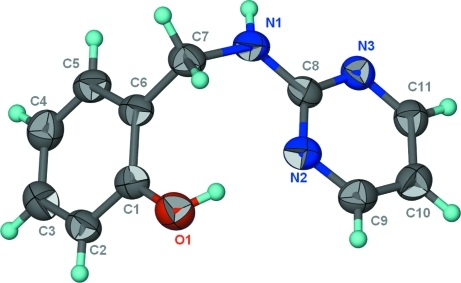
Thermal ellipsoid plot (Barbour, 2001) of C11H11N3O at the 50% probability level; hydrogen atoms are drawn as spheres of arbitrary radius.

## supplementary crystallographic information

### Comment

There are numerous studies on the Schiff bases derived by condensing salicyldehyde and an aromatic amine. In this study, the azomethine double-bond of *N*-salicylidene-2-aminopyrimidine (El-Haty *et al.*, 1990; Issa *et al.*, 2011; Shalabi & Abdel-Ghani, 1990) is reduced by sodium borohydride to yield the title secondary amine (Scheme I). The two aromatic rings at either ends of the –CH_2_–NH– link of C_11_H_11_N_3_O are twisted by 72.58 (8)°; the hydroxy substituent is hydrogen-bond donor to anone N atom of the pyrimidyl ring (Fig. 1). The other N atom of the pyrimidyl ring is hydrogen-bond acceptor to the amino group of an inversion-related molecule (Table 1).

### Experimental

A solution of 2-aminopyrimidine (1 mmol) and salicylaldehyde (1 mmol) in toluene (50 ml) was heated for 10 h. The solvent was removed under vacuum, and the residue was reduced in absolute methanol by sodium borohydride. Colorless crystals were obtained by recrystallization from methanol; yield 80%.

### Refinement

Carbon-bound H-atoms were placed in calculated positions (C–H 0.93–0.97 Å) and were included in the refinement in the riding model approximation, with U_iso_(H) = 1.2U_eq_(C). The amino and hydroxy H-atoms were located in a difference Fourier map, and were refined with distance restraints N–H 0.88±0.01 Å and O–H 0.84±0.01 Å; their temperature factors were refined.

### Crystal data

**Table:**

C_11_H_11_N_3_O	*F*(000) = 424
*M**_r_* = 201.23	*D*_x_ = 1.329 Mg m^−^^3^
Monoclinic, *P*2_1_/*n*	Mo *K*α radiation, λ = 0.71073 Å
Hall symbol: -P 2yn	Cell parameters from 6005 reflections
*a* = 5.8625 (4) Å	θ = 3.1–27.4°
*b* = 9.3610 (7) Å	µ = 0.09 mm^−^^1^
*c* = 18.4058 (13) Å	*T* = 293 K
β = 95.208 (2)°	Prism, colorless
*V* = 1005.92 (12) Å^3^	0.22 × 0.17 × 0.15 mm
*Z* = 4	

### Data collection

**Table:**

Rigaku R-AXIS RAPID IP diffractometer	2296 independent reflections
Radiation source: fine-focus sealed tube	1476 reflections with *I* > 2σ(*I*)
graphite	*R*_int_ = 0.030
ω scan	θ_max_ = 27.4°, θ_min_ = 3.1°
Absorption correction: multi-scan (*ABSCOR*; Higashi, 1995)	*h* = −7→6
*T*_min_ = 0.981, *T*_max_ = 0.987	*k* = −12→12
9626 measured reflections	*l* = −23→23

### Refinement

**Table:**

Refinement on *F*^2^	Primary atom site location: structure-invariant direct methods
Least-squares matrix: full	Secondary atom site location: difference Fourier map
*R*[*F*^2^ > 2σ(*F*^2^)] = 0.040	Hydrogen site location: inferred from neighbouring sites
*wR*(*F*^2^) = 0.149	H atoms treated by a mixture of independent and constrained refinement
*S* = 1.12	*w* = 1/[σ^2^(*F*_o_^2^) + (0.0796*P*)^2^ + 0.034*P*] where *P* = (*F*_o_^2^ + 2*F*_c_^2^)/3
2296 reflections	(Δ/σ)_max_ = 0.001
144 parameters	Δρ_max_ = 0.14 e Å^−^^3^
2 restraints	Δρ_min_ = −0.15 e Å^−^^3^

### Fractional atomic coordinates and isotropic or equivalent isotropic displacement parameters (Å^2^)

**Table:**

	*x*	*y*	*z*	*U*_iso_*/*U*_eq_	
O1	1.0476 (2)	0.91985 (13)	0.62203 (7)	0.0630 (4)	
N1	0.5979 (2)	0.67826 (16)	0.53868 (8)	0.0571 (4)	
N2	0.9251 (2)	0.77099 (14)	0.49494 (7)	0.0534 (4)	
N3	0.7438 (2)	0.55875 (14)	0.44465 (7)	0.0517 (4)	
C1	0.9836 (3)	0.82174 (16)	0.67131 (9)	0.0488 (4)	
C2	1.1341 (3)	0.79323 (18)	0.73237 (9)	0.0554 (4)	
H2	1.2718	0.8428	0.7393	0.066*	
C3	1.0819 (3)	0.69305 (19)	0.78229 (10)	0.0622 (5)	
H3	1.1850	0.6737	0.8225	0.075*	
C4	0.8756 (3)	0.62029 (19)	0.77307 (10)	0.0646 (5)	
H4	0.8405	0.5512	0.8066	0.078*	
C5	0.7224 (3)	0.65110 (18)	0.71357 (10)	0.0581 (4)	
H5	0.5827	0.6034	0.7081	0.070*	
C6	0.7718 (2)	0.75136 (16)	0.66180 (9)	0.0500 (4)	
C7	0.5997 (3)	0.78265 (19)	0.59729 (9)	0.0580 (4)	
H7A	0.4480	0.7868	0.6143	0.070*	
H7B	0.6329	0.8760	0.5779	0.070*	
C8	0.7604 (2)	0.66981 (16)	0.49207 (8)	0.0478 (4)	
C9	1.0801 (3)	0.75831 (19)	0.44642 (10)	0.0574 (4)	
H9	1.1954	0.8266	0.4466	0.069*	
C10	1.0780 (3)	0.65013 (19)	0.39651 (9)	0.0593 (5)	
H10	1.1880	0.6432	0.3633	0.071*	
C11	0.9034 (3)	0.55172 (19)	0.39818 (9)	0.0550 (4)	
H11	0.8973	0.4766	0.3650	0.066*	
H1O	1.000 (4)	0.889 (3)	0.5791 (8)	0.117 (9)*	
H1N	0.501 (3)	0.6073 (14)	0.5417 (10)	0.062 (5)*	

### Atomic displacement parameters (Å^2^)

**Table:**

	*U*^11^	*U*^22^	*U*^33^	*U*^12^	*U*^13^	*U*^23^
O1	0.0674 (8)	0.0543 (7)	0.0667 (8)	−0.0114 (5)	0.0032 (6)	0.0017 (6)
N1	0.0425 (7)	0.0658 (9)	0.0629 (8)	−0.0070 (6)	0.0040 (6)	−0.0127 (7)
N2	0.0488 (7)	0.0504 (8)	0.0601 (8)	−0.0022 (6)	0.0006 (6)	0.0040 (6)
N3	0.0495 (7)	0.0547 (8)	0.0500 (7)	0.0018 (6)	0.0006 (6)	−0.0008 (6)
C1	0.0492 (8)	0.0404 (8)	0.0572 (9)	0.0000 (6)	0.0070 (7)	−0.0069 (7)
C2	0.0488 (8)	0.0575 (10)	0.0590 (10)	−0.0035 (7)	−0.0001 (8)	−0.0112 (8)
C3	0.0656 (11)	0.0631 (11)	0.0563 (10)	0.0039 (8)	−0.0026 (8)	−0.0032 (8)
C4	0.0772 (12)	0.0569 (10)	0.0611 (10)	−0.0049 (9)	0.0134 (9)	0.0010 (8)
C5	0.0504 (9)	0.0557 (10)	0.0694 (11)	−0.0077 (7)	0.0120 (8)	−0.0116 (8)
C6	0.0427 (8)	0.0484 (9)	0.0591 (10)	0.0026 (6)	0.0064 (7)	−0.0118 (7)
C7	0.0446 (8)	0.0635 (10)	0.0652 (10)	0.0062 (7)	0.0016 (8)	−0.0130 (8)
C8	0.0426 (8)	0.0499 (9)	0.0494 (8)	0.0026 (6)	−0.0039 (7)	0.0025 (7)
C9	0.0523 (9)	0.0568 (10)	0.0629 (10)	−0.0025 (7)	0.0042 (8)	0.0124 (8)
C10	0.0572 (10)	0.0637 (11)	0.0581 (10)	0.0031 (8)	0.0100 (8)	0.0067 (8)
C11	0.0581 (9)	0.0561 (10)	0.0503 (9)	0.0067 (8)	0.0019 (8)	0.0012 (7)

### Geometric parameters (Å, °)

**Table:**

O1—C1	1.367 (2)	C3—H3	0.9300
O1—H1O	0.863 (10)	C4—C5	1.382 (3)
N1—C8	1.341 (2)	C4—H4	0.9300
N1—C7	1.455 (2)	C5—C6	1.387 (2)
N1—H1N	0.878 (9)	C5—H5	0.9300
N2—C9	1.336 (2)	C6—C7	1.515 (2)
N2—C8	1.350 (2)	C7—H7A	0.9700
N3—C11	1.325 (2)	C7—H7B	0.9700
N3—C8	1.3552 (19)	C9—C10	1.367 (2)
C1—C2	1.390 (2)	C9—H9	0.9300
C1—C6	1.402 (2)	C10—C11	1.380 (2)
C2—C3	1.367 (2)	C10—H10	0.9300
C2—H2	0.9300	C11—H11	0.9300
C3—C4	1.385 (3)		
			
C1—O1—H1O	107.4 (18)	C5—C6—C1	118.05 (15)
C8—N1—C7	123.78 (14)	C5—C6—C7	120.21 (15)
C8—N1—H1N	119.7 (12)	C1—C6—C7	121.74 (15)
C7—N1—H1N	115.0 (12)	N1—C7—C6	114.25 (13)
C9—N2—C8	116.03 (14)	N1—C7—H7A	108.7
C11—N3—C8	116.12 (14)	C6—C7—H7A	108.7
O1—C1—C2	118.23 (14)	N1—C7—H7B	108.7
O1—C1—C6	121.74 (14)	C6—C7—H7B	108.7
C2—C1—C6	120.03 (15)	H7A—C7—H7B	107.6
C3—C2—C1	120.70 (15)	N1—C8—N2	118.73 (14)
C3—C2—H2	119.7	N1—C8—N3	116.33 (14)
C1—C2—H2	119.7	N2—C8—N3	124.93 (15)
C2—C3—C4	120.10 (16)	N2—C9—C10	123.37 (16)
C2—C3—H3	120.0	N2—C9—H9	118.3
C4—C3—H3	120.0	C10—C9—H9	118.3
C5—C4—C3	119.47 (17)	C9—C10—C11	116.19 (16)
C5—C4—H4	120.3	C9—C10—H10	121.9
C3—C4—H4	120.3	C11—C10—H10	121.9
C4—C5—C6	121.60 (16)	N3—C11—C10	123.36 (16)
C4—C5—H5	119.2	N3—C11—H11	118.3
C6—C5—H5	119.2	C10—C11—H11	118.3
			
O1—C1—C2—C3	−178.05 (15)	C5—C6—C7—N1	−79.8 (2)
C6—C1—C2—C3	2.4 (2)	C1—C6—C7—N1	100.33 (18)
C1—C2—C3—C4	−1.1 (3)	C7—N1—C8—N2	−6.1 (2)
C2—C3—C4—C5	−0.8 (3)	C7—N1—C8—N3	174.43 (14)
C3—C4—C5—C6	1.4 (3)	C9—N2—C8—N1	−178.93 (13)
C4—C5—C6—C1	0.0 (2)	C9—N2—C8—N3	0.4 (2)
C4—C5—C6—C7	−179.92 (15)	C11—N3—C8—N1	179.28 (13)
O1—C1—C6—C5	178.65 (14)	C11—N3—C8—N2	−0.1 (2)
C2—C1—C6—C5	−1.8 (2)	C8—N2—C9—C10	−0.5 (2)
O1—C1—C6—C7	−1.5 (2)	N2—C9—C10—C11	0.2 (2)
C2—C1—C6—C7	178.06 (14)	C8—N3—C11—C10	−0.2 (2)
C8—N1—C7—C6	−74.7 (2)	C9—C10—C11—N3	0.2 (2)

### Hydrogen-bond geometry (Å, °)

**Table:**

*D*—H···*A*	*D*—H	H···*A*	*D*···*A*	*D*—H···*A*
O1—H1o···N2	0.86 (1)	1.92 (1)	2.761 (2)	164 (2)
N1—H1n···N3^i^	0.88 (1)	2.15 (1)	3.023 (2)	176 (2)

Symmetry codes: (i) −*x*+1, −*y*+1, −*z*+1.
